# Curcumin as an antidote to atrazine on estrogen homeostasis and beyond: mechanistic insights from a targeted literature review

**DOI:** 10.3389/fendo.2025.1553465

**Published:** 2025-07-23

**Authors:** Patrick J. Wright, Jade Ilali, Pengli Bu

**Affiliations:** Department of Pharmaceutical Sciences, College of Pharmacy and Health Sciences, St. John’s University, Queens, NY, United States

**Keywords:** atrazine, breast cancer, curcumin, CYP19A1, CYP3A4, EGF signaling, estrogen homeostasis

## Abstract

As the most common cancer in women globally, breast cancer poses a significant public health concern. More concerning is its rising incidence rates in certain areas of the world, including Australia, New Zealand, Western Europe, and North America. Exposure to environmental endocrine-disrupting chemicals may play a role. One such chemical is atrazine (ATZ), a man-made herbicide highly prevalent in the environment and detectable in drinking water, with reported levels ranging from 0.026 to 1.29 micrograms per liter (µg/L) in surface waters in the United States. During the development of breast cancer, many factors are involved, in particular the female sex hormone estrogen. Estrogen signaling fuels the proliferation and migration of estrogen receptor (ER)-positive breast cancer. The current review presents multiple lines of qualitative evidence from *in vitro*, *in vivo*, and epidemiological studies connecting ATZ exposure to processes important for breast cancer development. Specifically, ATZ’s stimulatory effect on breast cancer is mediated, at least partially, through enhanced CYP19A1 activity, the key enzyme converting testosterone to estradiol. ATZ stimulates CYP19A1 activity via parallel pathways, as evidenced by *in vitro* studies, potentially leading to elevated estradiol levels and estrogen signaling, which would then drive the development of ER-positive breast cancers. Beyond estrogen signaling, ATZ taps into the epidermal growth factor (EGF) signaling pathway to stimulate uncontrolled proliferation in human cell lines. We then show how curcumin, a phytochemical in turmeric, may counteract ATZ’s effect on the aforementioned processes. Once curcumin passes through the ADME process and becomes available in the human body, curcumin may possess effects to counter ATZ’s toxicity. Curcumin induces CYP3A4, as demonstrated by *in vitro* and *in vivo* studies, which catalyzes the degradation of steroid hormones, including estrogen. Curcumin downregulates the basal level of CYP19A1 in human cell lines via miRNA-125a and estrogen-related receptor alpha (ERRα), indicating an ability to dampen estrogen signaling. In addition, curcumin has been shown to inhibit the EGF receptor in human cell lines, thus blocking the EGF signaling cascade at the receptor level. Furthermore, curcumin may reduce ATZ’s overall bioavailability. ATZ and its metabolites undergo glutathione (GSH) conjugation followed by renal excretion. Curcumin helps maintain the GSH pool and activates glutathione-S-transferase (GST) in rats, thereby potentially facilitating the detoxification and elimination of ATZ. In conclusion, we propose that curcumin’s ability to induce CYP3A4, suppress CYP19A1, inhibit EGF signaling, and promote detoxification and elimination of ATZ makes curcumin a promising candidate for a mechanism-based antidote to ATZ toxicity.

## Introduction

Breast cancer, a disease of pivotal significance to women’s health, is the most common cancer in women globally (1). Breast cancer is also the second leading cause of cancer-related death in women in the United States (2). With advancements in chemo- and immunotherapy for cancer treatment, the mortality rate of breast cancer is decreasing (3). However, incidence rates of breast cancer are increasing in regions including Australia, New Zealand, Western Europe, and North America (3). Environmental contaminants with endocrine-disrupting properties, i.e., the so-called endocrine-disrupting chemicals, have been proposed to be possible culprits contributing to this rising rate (4, 5). One relevant contaminant is atrazine (ATZ), the second-most used pesticide in the United States (6). ATZ is a synthetic herbicide commonly applied to many crops, including corn, sorghum, and sugarcane (7). ATZ was banned in the European Union in 2003 due to concerns of water contamination, but remains in use in the United States (8). In July 2024, the U.S. Environmental Protection Agency (EPA) increased acceptable water levels of ATZ from 3.4 micrograms per liter (µg/L) to 9.7 µg/L (9). As a result, the widespread use of ATZ leads to its accumulation in the environment and its detection in river systems, groundwater, and drinking water (5, 7, 8, 10). Specifically, reported levels of ATZ ranged from 0.026 to 1.29 µg/L in the Chesapeake Bay surface water system, which is significant to the mid-Atlantic region of the United States, where agriculture is a major industry (11). Therefore, obtaining a better understanding of how chronic exposure to ATZ could link to breast cancer is of significant value to public health.

Breast cancer is characterized by uncontrolled cell growth and proliferation in the breast, usually in the lobules or ducts (12). Breast cancers are often carcinomas, which are tumors that start in epithelial cells and later acquire the ability to metastasize to secondary sites (13). During the carcinogenesis of breast cancer, many factors and pathways are involved, including estrogen signaling, which plays a key role in the progression of some types of breast cancer. Estrogen is the principal female sex hormone essential for the establishment of female sexual characteristics, particularly for breast development (14). Clinically, breast cancer is categorized by the presence of certain proteins in the cancer cells, such as the estrogen receptor (ER) (15). If ER is present in breast cancer cells, such breast cancer is categorized as ER-positive (15). In ER-positive breast cancer, elevated estrogen signaling is crucial in fueling the growth and metastasis of tumor cells (15). Abnormally elevated estrogen binding to ER leads to increased estrogen signaling, driving cell proliferation and migration (15). By employing a targeted literature review approach, we present evidence and associated mechanisms of how ATZ stimulates estrogen signaling in various experimental settings and epidemiological studies, potentially contributing to increased breast cancer risk.

Curcumin is a bright yellow phytochemical abundantly present in turmeric, a perennial plant of the ginger family. Widely recognized as an herbal remedy, curcumin has long been used in human history for an array of conditions, including arthritis, metabolic syndrome, pain, and, more recently, oxidative stress related to Alzheimer’s disease (16–18). Curcumin’s low bioavailability may limit its overall effects on the body (19, 20). Curcumin is poorly absorbed in the body and metabolized rapidly (19, 20). Such low bioavailability diminishes curcumin’s effectiveness in the human body (20). This limitation is likely a major factor preventing curcumin’s clinical approval as a treatment for medical conditions, despite evidence supporting its safety at doses as high as 12 grams per day (20). As a result, low bioavailability may hinder curcumin’s usefulness in combating ATZ-induced toxicity as detailed in the discussion below.

In the current review, we first present qualitative evidence connecting ATZ exposure to breast cancer processes and then provide insights on how curcumin may serve as a mechanism-based antidote for ATZ via antagonizing ATZ’s effect on estrogen homeostasis and beyond. **Table 1** compares ATZ and curcumin in their key physicochemical properties (7, 8, 16, 17, 21–24).

**Table 1:** Comparison between ATZ and curcumin: physicochemical properties and more.

Property	Atrazine	Curcumin
Ball-and-Stick Structure	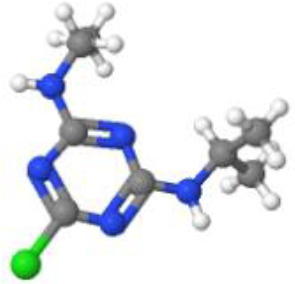	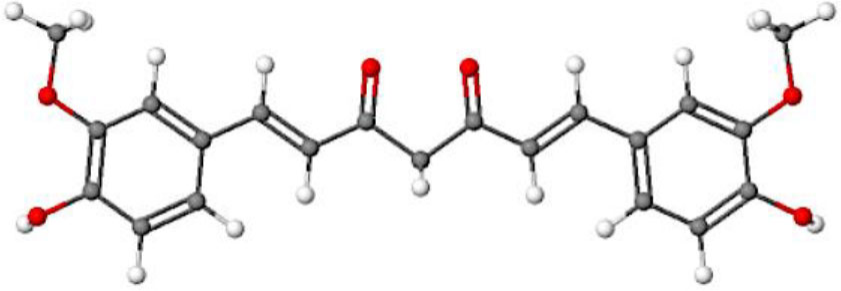
Line Formula Structure	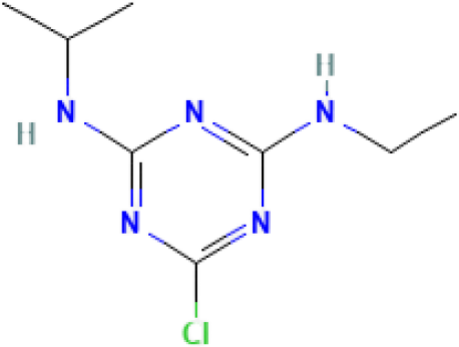	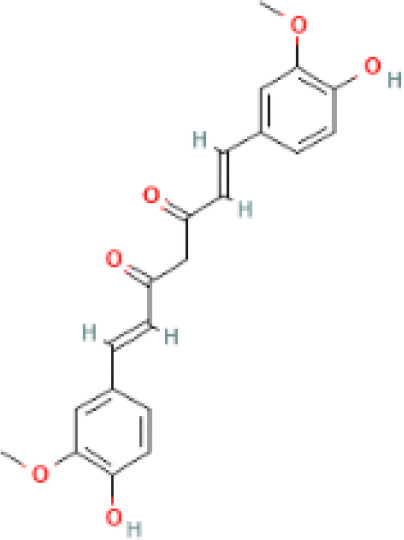
Formula	C_8_H_14_ClN_5_	C_21_H_20_O_6_
Molecular Weight	215.683 g/mol	368.39 g/mol
Melting Point	173 ˚C	183 ˚C
Boiling Point	N/A	N/A
Water Solubility	35 mg/L	7.48 mg/L
Natural or Man-Made	Man-Made	Natural Substance or Synthetic
Source	Synthesized in Lab	Turmeric
Utility	Herbicide	Health Supplement
Main Route of Exposure	Contaminated Drinking Water	Oral Ingestion
Relevant Toxicity Information	Reproductive Organs	None
Historical Information	Banned in the EU since 2003, and controversial in the United States	Used for thousands of years
Research Focus	Toxicity to the endocrine system and reproductive system	Relevance to steroid hormone metabolism pathway
Research Highlights	Able to increase steroidogenesis, particularly estrogen production	Induction of CYP3A4

## Methods

We conducted initial searches on PubMed and Google Scholar to gain an understanding of the existing literature on ATZ and curcumin. We then expanded our search to additional databases, including the Human Metabolome Database (HMDB), the Kyoto Encyclopedia of Genes and Genomes (KEGG), and the National Center for Biotechnology Information (NCBI) Gene database. The papers produced by the NCBI Gene database searches are all indexed in PubMed.

We searched for “atrazine” in the NCBI Gene database with the *Homo sapiens* filter. Four genes were returned: CYP19A1, NR5A1 (also known as steroidogenic factor-1 (SF-1)), ATP5ME, and NPM1P14. We then searched the bibliographies (PubMed papers) of each gene. We screened the bibliography paper titles for “atrazine”, “ATR”, or “ATZ” to identify any reported connection between each gene and ATZ. Specifically, we screened the titles of 833 papers on CYP19A1, 288 papers on SF-1, 74 papers on ATP5ME, and 2 papers on NPM1P14. We found 1 paper for CYP19A1 + ATZ, 2 papers for SF-1 + ATZ, 1 paper for ATP5ME + ATZ, and 1 paper for NPM1P14 + ATZ. The results suggested to us a possible connection between ATZ and estrogen homeostasis encompassing CYP19A1 and SF-1. We then searched the NCBI Gene database for “curcumin”, again with the *Homo sapiens* filter. This produced 232 genes. However, there was no overlap with the four genes (CYP19A1, SF-1, ATP5ME, NPM1P14) returned from the “atrazine” search. We then refined our search by adding the keyword “estrogen” (i.e., curcumin + estrogen + *Homo sapiens*), reducing the list to 128 genes. We further refined our search to only include genes/proteins with “estrogen” in their name, which produced four genes: ESR2 (also called estrogen receptor beta (ERβ)), TRPM2, HSPB1, and ERRα. Next, we examined the bibliographies (PubMed papers) associated with each gene, screening paper titles for the words “curcumin”, “steroid”, and/or “estrogen”. We screened the titles of 1,275 papers on ESR2, 166 papers on TRPM2, 797 papers on HSPB1, and 228 papers on ERRα. We found 1 paper on ESR2, 4 papers on TRPM2, 9 papers on HSPB1, and 4 papers on ERRα featuring our keyword(s) in the titles. ERRα was considered a highly relevant gene to the estrogen homeostasis theme, and therefore, the 4 papers on ERRα were selected. Later, when we revisited the 128 genes associated with the “curcumin” search, we identified CYP3A4 as another gene highly relevant to the estrogen homeostasis theme due to its role in estrogen turnover.

Given the relevance of breast cancer in women worldwide and the key role estrogen signaling plays in driving the development of breast cancer, we searched “atrazine”, “curcumin”, and “breast cancer” in PubMed and Google Scholar. We identified several highly relevant papers encompassing *in vitro*, *in vivo*, and epidemiological studies. We thoroughly reviewed the papers yielded in the above searches and selected the ones most relevant to the estrogen homeostasis theme, and also reviewed their references within. We then extracted the most relevant findings, performed secondary analysis, and constructed a table and figures that are presented in this review.

### ATZ promotes breast cancer development

**Figure 1:**
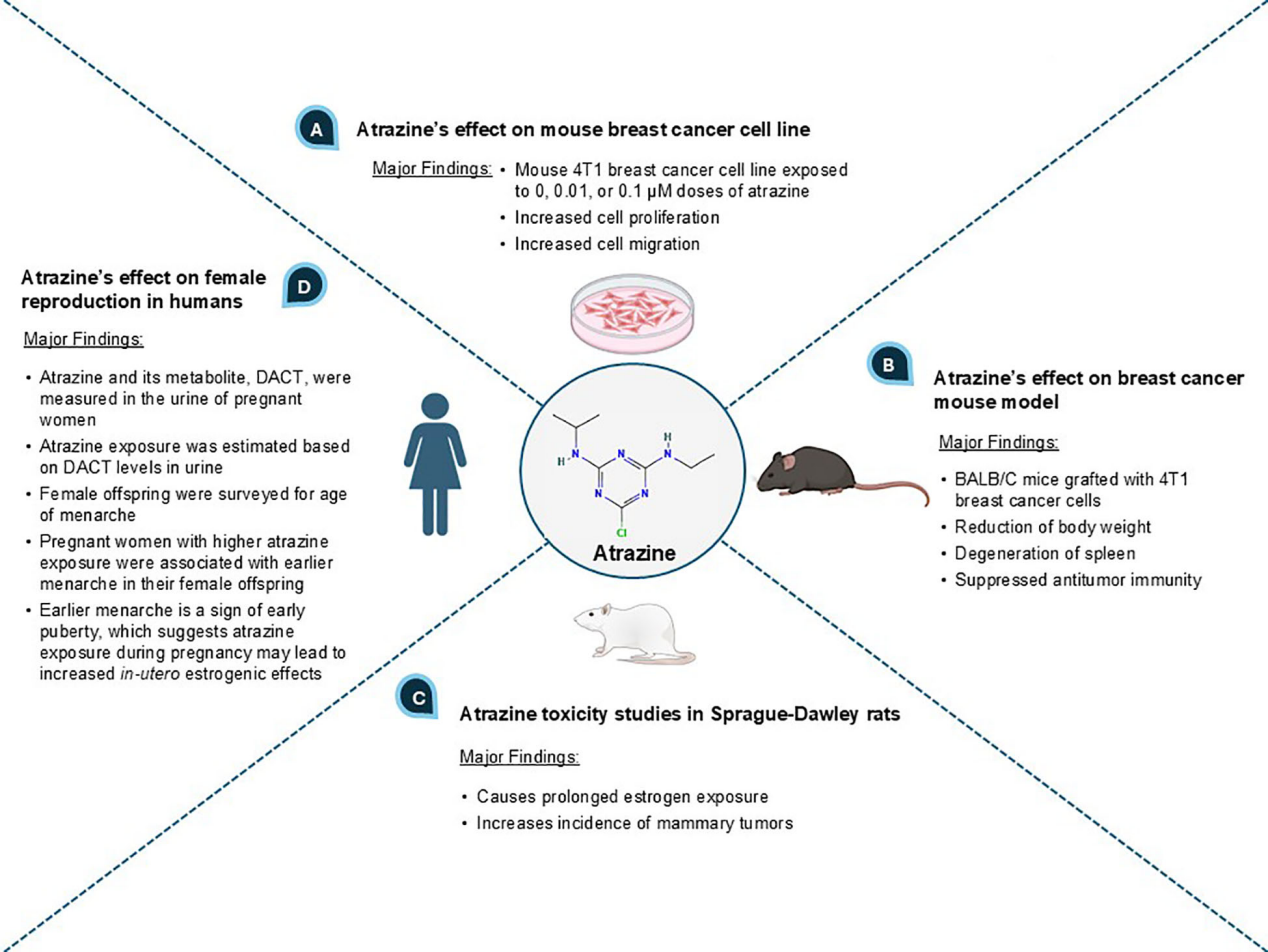
An overview of ATZ’s effects on breast cancer and estrogen effect.

An initial search was conducted on Google Scholar using the term “atrazine and breast cancer”. In the returned search results, we screened the top ten hits (and references within) encompassing *in vitro*, *in vivo*, and epidemiological studies and selected the most relevant papers reporting on the relationship between ATZ exposure, estrogenic effect, and breast cancer (25, 26). We then refined our search terms, focusing on potential mechanisms, and searched the PubMed database, where we identified relevant mechanistic studies that further supported a connection between ATZ and breast cancer. The key evidence showing a correlative relationship between ATZ exposure, estrogenic effect, and breast cancer processes is presented in **Figure 1**.

When the mouse breast tumor cell line 4T1 was exposed to ATZ in doses of 0.01 and 0.1 micromolar (µM), these cells showed increased cell proliferation and migration compared to the control group, which demonstrated ATZ’s ability to stimulate breast cancer cells *in vitro* (26) (**Figure 1A**). BALB/C mice with 4T1 breast cancer cell grafts were orally administered ATZ dissolved in maize oil at doses of 20 and 100 mg/kg body weight for 28 days (26) (**Figure 1B**). The oral administration mimics human exposure to ATZ, which is typically present in drinking water as a result of ATZ accumulation in surface water, groundwater, and estuarine systems (7, 11). ATZ-treated mice showed spleen degeneration, reduced body weight, and suppressed antitumor immunity in a dose-dependent manner, demonstrating that ATZ exposure promoted mammary tumor progression *in vivo* (26) (**Figure 1B**). In another preclinical study, ATZ mixed in chow was given to Sprague-Dawley rats at doses ranging from 10 to 1000 parts per million (ppm), which converts to approximately 46 to 4,600 µM (27). Results showed increased amounts of mammary tumors compared to non-exposed rats, suggesting a potential causal relationship (27) (**Figure 1C**).

Human breast cancer cell line MCF-7 was treated with ATZ at 100 parts per billion (ppb), which resulted in statistically significant alterations in cell morphology and protein expression profile (28). In particular, under-expression of proteins relevant to DNA maintenance, such as ubiquitin-conjugating enzyme E2 N and superoxide dismutase 1, could suggest a causal relationship between ATZ and cancer progression (28). In another study, MCF-7 cells exposed to environmentally relevant concentrations of ATZ displayed increased cell proliferation (29). MCF-10A, a normal human breast epithelial cell line, also displayed increased cell proliferation upon exposure to the same concentration of ATZ, though this proliferation was less than that observed in MCF-7 (29). Such evidence suggests ATZ’s potential involvement in both the onset of breast cancer and the progression of breast cancer (29). MCF-10A cells also exhibited activation of DNA damage repair response, specifically the ATR-Chk1 pathway, upon exposure to ATZ (30). This implies that ATZ has the capability to damage DNA, which could link ATZ to an increased risk of breast cancer initiation. T-47D, another human breast cancer cell line, was exposed to environmentally relevant ATZ concentrations and showed upregulation of CYP19A1, GATA-3, GAT-4, and c-Myc (31). The author speculated that such changes at developmental stages, including prenatal, might increase the risk of breast cancer development (31).

A longitudinal epidemiological study in humans analyzed the levels of diaminochlorotriazine (DACT), a metabolite of ATZ, in 3,938 urine samples of pregnant women from southwestern England to estimate ATZ exposure (32) (**Figure 1D**). These samples were collected from women with expected delivery dates ranging from April 1^st^, 1991 to December 31^st^, 1992 (32). From 1990 to 1992, 10,000 hectares (ha), or 100,000,000 square meters (m^2^), in southwestern England received approximately 17,000 kg of ATZ annually (32). An inverse correlation between ATZ exposure levels in pregnant women and the age of menarche in their female offspring was established (i.e., the greater the ATZ exposure, the earlier the age of menarche) (32). This correlation was statistically significant for the subset of subjects with complete data who had DACT levels greater than or equal to the median (32). Clinically, early menarche is considered a marker of early puberty and a risk factor for developing breast cancer (33–35). Since ATZ exposure to pregnant women was correlated with early menarche in their female offspring, *in-utero* ATZ exposure may increase the risk of female offspring developing breast cancer later in life (33–35). We speculate that the underlying mechanism may be increased *in-utero* estrogenic effects caused by maternal ATZ exposure (see later sections on how ATZ may increase estradiol biosynthesis via CYP19A1 induction to augment estrogen availability and intensify estrogenic effects in the developing fetus). The *in-utero* exposure to other endocrine-disrupting chemicals, such as polybrominated biphenyls (PBBs) and dichlorodiphenyldichloroethylene (DDE), and their correlations with earlier age of menarche, have also been reported (36, 37). Our hypothesis, focusing on the elevated estrogen signaling and effect as the underlying connection between early menarche age and *in-utero* ATZ exposure, may point to a promising direction for future research.

### Curcumin may serve as an antidote to ATZ via distinct mechanisms

#### Curcumin counters ATZ’s stimulation of estrogen signaling

**Figure 2:**
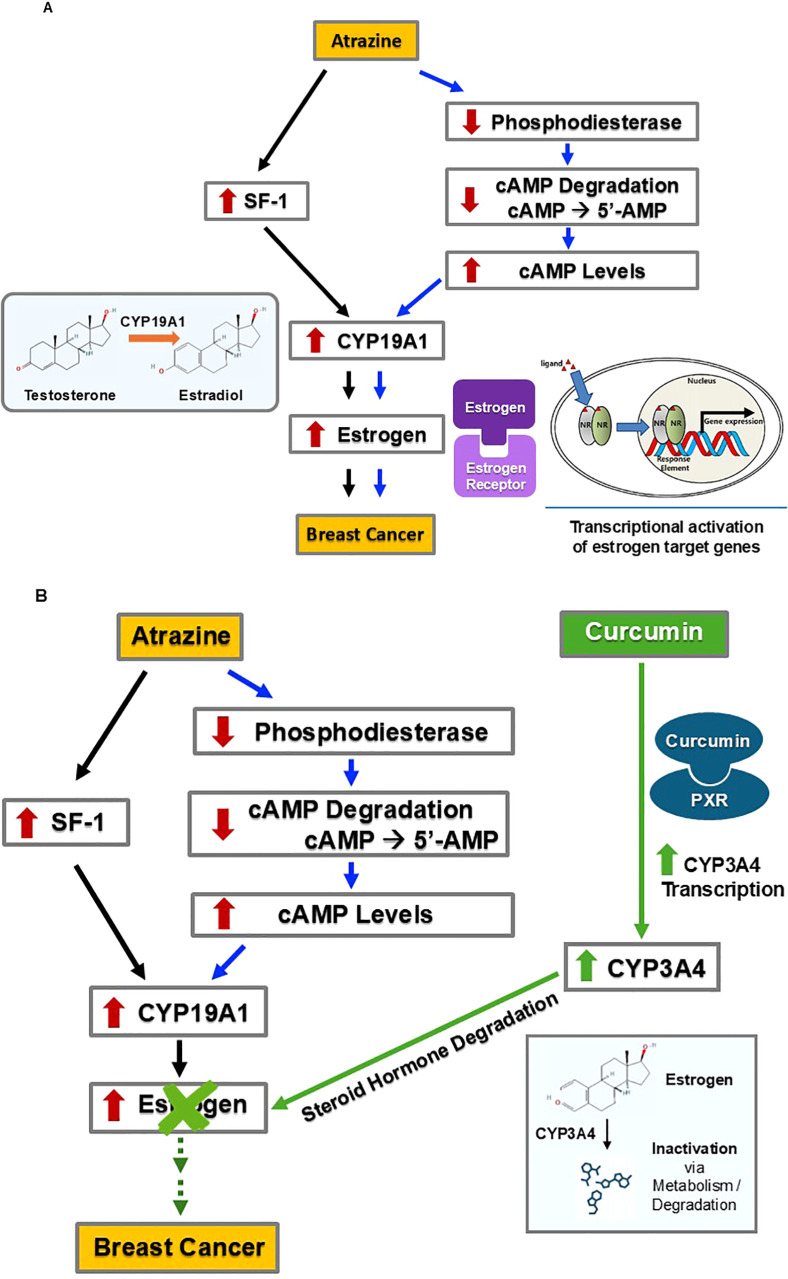
Through promoting estrogen degradation, curcumin may counteract ATZ’s effect on breast cancer development. **(A)** ATZ may promote breast cancer development via estrogen-dependent mechanisms. **(B)** Curcumin may antagonize ATZ’s effect by stimulating estrogen degradation.

ATZ’s stimulatory effect on breast cancer is mediated, at least in part, by elevating estrogen levels, thereby enhancing estrogen signaling intensity (**Figure 2A**). ATZ increases estrogen levels via two parallel pathways, both converging on the induction of CYP19A1 expression. CYP19A1, also known as aromatase, is an enzyme that catalyzes the last step in estradiol biosynthesis, i.e., the conversion of testosterone to estradiol (38, 39) (**Figure 2A**). Therefore, the activity of CYP19A1 is essential in determining the amount of estradiol produced and the ratio between testosterone and estradiol. In one pathway, ATZ activates Steroidogenic Factor-1 (SF-1) by direct binding, as observed in H295R human adrenocortical carcinoma cells (40) (**Figure 2A** left part). In the presence of SF-1-dependent ArPII promoter, SF-1 activation led to the induction of CYP19A1 expression and subsequent activity (40). Human ovarian granulosa-like KGN tumor cells, which lack SF-1, were unresponsive upon exposure to ATZ until SF-1 was added exogenously, confirming that SF-1 is necessary for CYP19A1 induction (41). In the parallel pathway, ATZ inhibits phosphodiesterase, as seen in 51 day old Wistar rat Leydig cells and 50 day old Sprague-Dawley rat anterior pituitary cells, as well as mouse gonadotroph pituitary cell line L-βT2 and rat lacto-somatotroph pituitary cell line GH_3_ (42). Additionally, ATZ directly bound and inhibited phosphodiesterase at a minimum concentration of 5 nanomolar (nM) – which is a concentration comparable to environmental ATZ exposure levels – in a cell-free assay (43) (**Figure 2A** right part). Phosphodiesterase hydrolyzes cyclic AMP (cAMP) to 5’-AMP, and by inhibiting phosphodiesterase, ATZ blocked the hydrolysis of cAMP to 5’-AMP, resulting in increased cAMP levels (42, 43). By extrapolating from the above *in vitro* studies, we conclude that ATZ exposure would lead to elevated cAMP levels due to its inhibition of phosphodiesterase. cAMP is a known inducer of CYP19A1 activity, as it activates Protein Kinase A (PKA) which in turn stimulates the CYP19A1 promoter (44). We speculate that increased intracellular cAMP levels caused by ATZ will lead to increased CYP19A1 activity. Therefore, ATZ can activate CYP19A1 activity via two parallel pathways, and the elevated activity of CYP19A1 leads to increased estradiol levels, which then contributes to a greater risk of breast cancer.

Curcumin fits in this picture by increasing the degradation of estrogen, thus counteracting ATZ’s effects (**Figure 2B**). Curcumin induced CYP3A4 in multiple models, including HepG2 cells (45), Sprague-Dawley rats (46), and ACI rats (47). HepG2 human hepatoma cells treated with curcumin (between concentrations of 1 µM and 50 µM) showed a dose-dependent increase in CYP3A4 transcription (45). Additionally, curcumin activated pregnane X receptor (PXR) in human wild-type hepatic progenitor HepaRG cells, primary human hepatocytes, human colon adenocarcinoma LS180 cells, and in animal models including mice and largemouth bass, as evidenced either by induction of PXR target genes (such as CYP3A4) or induction of reporter genes in reporter gene assays (45, 48–51). Furthermore, in the absence of PXR, curcumin failed to induce CYP3A4 mRNA (51). These results led us to hypothesize a mechanism responsible for the above observation: curcumin binds to PXR as an agonist (i.e., an activator or stimulator), and this agonist-receptor complex in turn induces CYP3A4 expression. In male Sprague-Dawley rats, administration of curcumin by gastric gavage at doses of 50 mg/1.0 mL/kg and 100 mg/2.0 mL/kg produced significant increases in CYP3A4 activity based on significantly decreased concentrations of a CYP3A4 substrate (46). In female ACI rats, curcumin administered via either a subcutaneous grafted implant (varying doses over 90 days) or oral administration (approximately 5 mg/kg per day over 90 days), led to activation of CYP3A4 (47). Subcutaneous administration appeared more effective, as it significantly increased CYP3A4 activity after 25 days, while oral administration led to a marked increase in CYP3A4 activity after 4 days of treatment, but returned to normal levels at 12 days of treatment (47). CYP3A4 catalyzes the degradation of steroid hormones, including estrogen (52, 53). Increased CYP3A4 activity following curcumin administration would enhance estrogen degradation. It is worth noting that hepatic CYP3A4 induction can help eliminate circulating estrogens, but its impact on local estrogen levels (i.e., estrogens produced in peripheral tissues, including the breast, via local aromatization and/or deconjugation) is likely to be limited (54). In summary, it seems possible that curcumin could counteract the increased circulating estrogen levels caused by ATZ and, therefore, may mitigate the potential ATZ-induced risk of breast cancer.

#### Curcumin may reduce CYP19A1 expression levels by suppressing ERRα, a mechanism independent of ATZ

**Figure 3:**
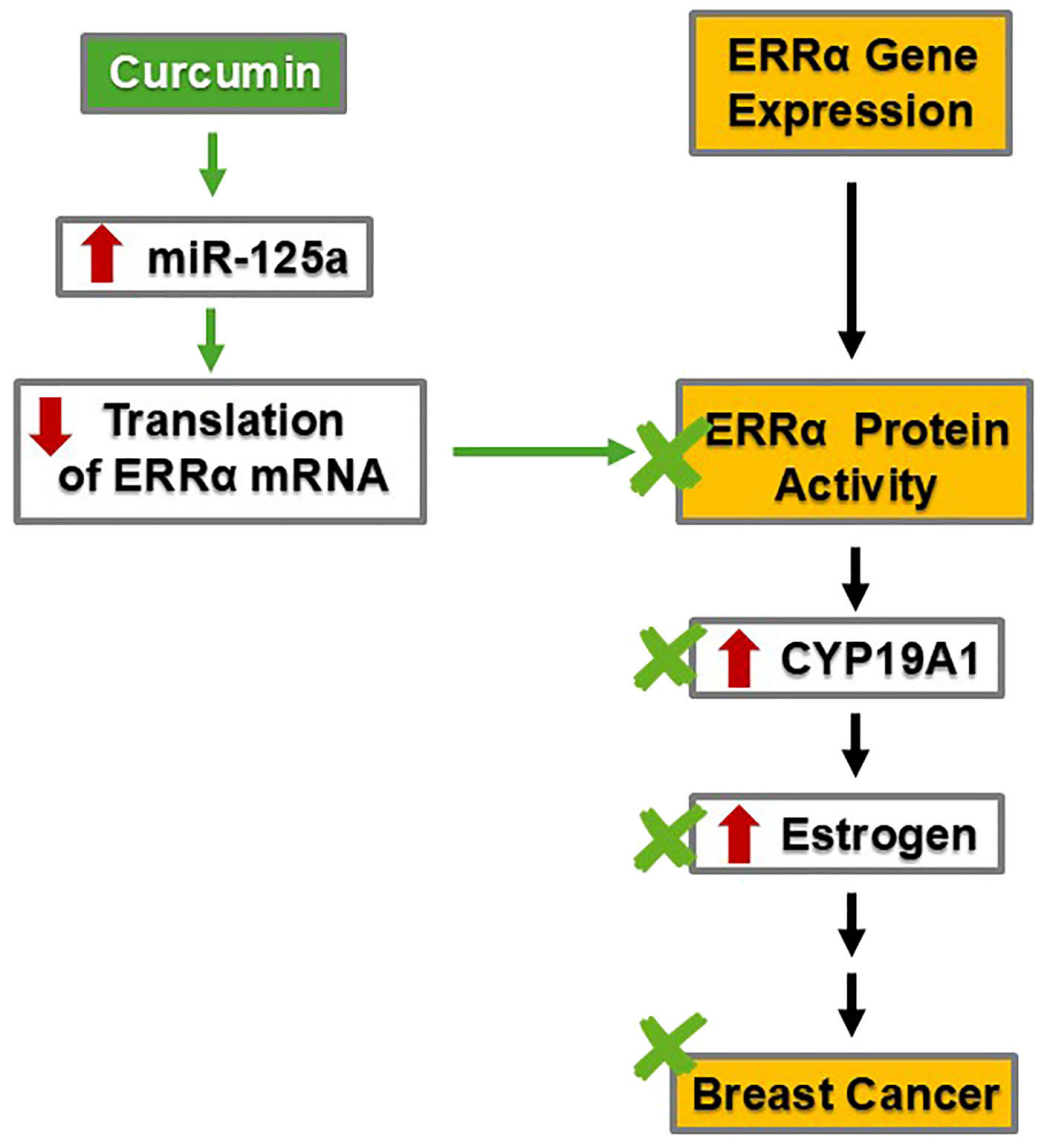
By decreasing the protein levels of ERRα and, subsequently, CYP19A1, curcumin may inhibit estrogen synthesis and reduce estrogen levels, thereby mitigating breast cancer risk.

As previously mentioned, CYP19A1 plays a critical role in the last step of estradiol biosynthesis. It is thus not surprising that multiple layers of regulation are in place to fine-tune CYP19A1 activity. In addition to its regulation by SF-1 and cAMP signaling, CYP19A1 is also a target of estrogen-related receptor alpha (ERRα) (55) (**Figure 3**).

ERRα is a transcription factor relevant to breast cancer development. ERRα is expressed in multiple human breast cancer cell lines, including ZR75.1, MDA-MB-231, HS587T, BT549, SK-BR-3, T47D, MCF-7, MDA-MB-448, and BT474 (56). *In vitro* experiments using human breast adenocarcinoma SK-BR-3 and human breast tumor fibroblast cell line WS3TF showed that ERRα increased the expression of CYP19A1 by binding to a silencer element (i.e., a type of regulatory DNA sequence) named S1, which allowed for promoter I.3 to increase CYP19A1 transcription, resulting in increased activity (55). Subsequently, increased CYP19A1 activity led to elevated estradiol levels (38, 39) (**Figures 2A**, **3**). This phenomenon and associated mechanism may be of tissue-specific nature; nevertheless, the two cell lines employed in the study are of particular relevance to breast cancer development.

By suppressing ERRα, curcumin mitigates breast cancer risk (**Figure 3**). Specifically, curcumin can stimulate the transcription of microRNA-125a (miRNA-125a), a small RNA species capable of regulating multiple targets post-transcriptionally, including translational suppression of ERRα (57). In human osteosarcoma cell lines U2OS and MG63, treatment with 20 µM curcumin increased the transcription and activity of miR-125a, which subsequently suppressed the translation of ERRα mRNA (57). The decrease in ERRα protein and activity would ensue. If this mechanism also holds true in the breast tissue, curcumin will suppress ERRα, resulting in less induction of CYP19A1 and subsequently less estradiol production. Through suppressing ERRα, curcumin could dampen estrogen signaling intensity and thus mitigate the risk of estrogen-dependent breast cancer in a more general setting.

#### Curcumin may offset ATZ’s potential breast cancer-progressive effects via the EGF signaling cascade

**Figure 4:**
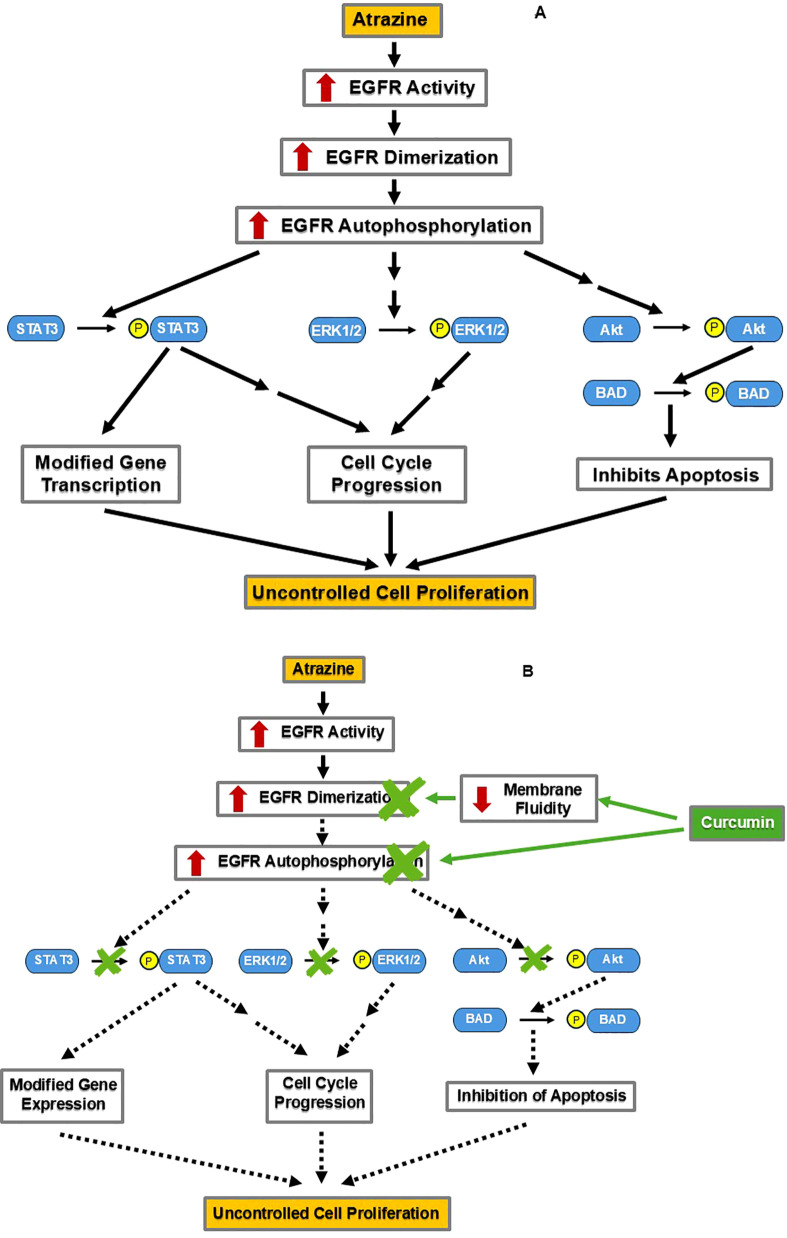
By decreasing EGFR activation, curcumin may counteract ATZ’s stimulation of cellular proliferation. **(A)** ATZ may promote the growth of breast cancer cells via the estrogen-independent EGF signaling pathway. **(B)** Curcumin may reduce ATZ’s stimulation of EGF signaling by inhibiting EGFR activation.

The mitogen epidermal growth factor (EGF) and its associated signaling cascade are essential in both normal development and the progression of many malignancies (58). Upon activation, the receptor of EGF (EGFR) dimerizes and undergoes autophosphorylation, initiating a cascade of signaling events (59) (**Figure 4A**). EGFR activation leads to the phosphorylation and activation of downstream enzymes: STAT3, ERK1/2, and Akt (59) (**Figure 4A**). Phosphorylated STAT3 directly modifies gene expression important for normal development and malignancies (59). Target genes of STAT3 include Bcl-2, an antiapoptotic gene, and IGFBP5, a gene involved in mammary tissue apoptosis (60). Phosphorylated STAT3 also indirectly stimulates cell cycle progression (59). Phosphorylated ERK1/2 indirectly increases cell cycle progression (59). Phosphorylated Akt phosphorylates and activates BAD, a protein that inhibits apoptosis (59). Collectively, these downstream effects contribute to cell proliferation, which is needed for normal development but can also be hijacked during cancer progression.

In addition to affecting estrogen signaling, ATZ also influences EGF signaling to promote uncontrolled cell proliferation. In a study employing two human ovarian cancer cell lines, namely BG-1 and 2008, when ATZ and the EGFR inhibitor AG1478 (AG) were co-present, the induction of c-Fos (a known ATZ effect) was prevented (61). Furthermore, the statistically significant stimulation of the proliferation of BG-1 and 2008 cells by ATZ-alone treatment was diminished in the ATZ+AG co-treatment group, suggesting that the EGFR is involved in mediating ATZ’s effects (61). This evidence supports the hypothesis that ATZ taps into the EGF signaling pathway to promote the uncontrolled proliferation of cancer cells (61).

Curcumin comes into play by inhibiting the EGF signaling pathway via distinct mechanisms (**Figure 4B**). One mechanism occurs at the cell surface and involves the EGFR protein (62). In a preclinical study, human oral squamous carcinoma cell line A-431 treated with curcumin showed disrupted lipid fluidity and increased rigidity of the cell membrane, which also reduced dimerization and autophosphorylation of the EGFR (62). Blocking of EGFR autophosphorylation would prevent downstream phosphorylation of STAT3, ERK1/2, and Akt, a consequence of the inhibition at the receptor level (**Figure 4B**). Indeed, curcumin administration inhibited STAT3, ERK1/2, and Akt phosphorylation in human oral squamous cell carcinoma cell line SCC-25 and decreased cell proliferation and tumor invasion in a dose-dependent manner (63). These findings demonstrate curcumin’s inhibition of the EGF pathway in certain cell lines. If curcumin can also inhibit the EGF pathway in the breast tissue, this would support its utility in managing EGF signaling-driven breast tumorigenesis, either triggered by ATZ or by other stimuli (**Figure 4B**).

#### Curcumin stimulates ATZ metabolism and excretion

**Figure 5:**
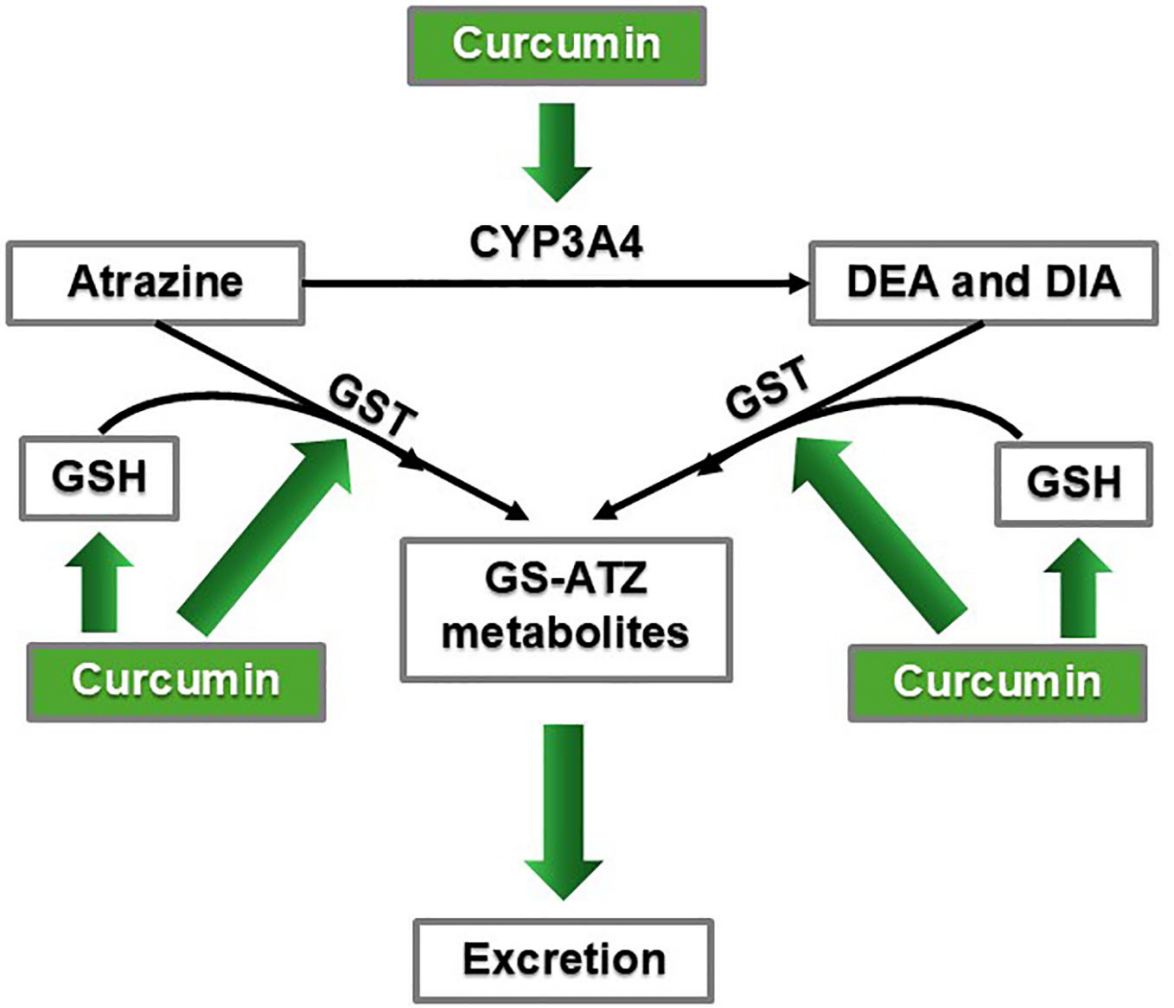
Curcumin may promote ATZ metabolism and excretion via CYP3A4 induction and enhanced GSH conjugation mechanisms.

Curcumin may play a role in determining ATZ bioavailability in the body. We constructed a summary figure to show how curcumin may potentially speed up the metabolism of ATZ (46, 64–67) (**Figure 5**). Based on the available studies, we propose that curcumin administration should be able to increase the metabolism of ATZ. ATZ is metabolized by CYP3A4 into two metabolites: desethylatrazine (DEA) and desisopropyl atrazine (DIA) in human liver microsomes and human liver S9 fractions (66). The same ATZ metabolites were also detected in raw (spring water) and finished water (tap and bottled water) (68), but their endocrine-disrupting properties remain to be fully understood (69). Multiple studies reported that CYP3A4 is induced by curcumin (45–47). Therefore, curcumin administration should increase CYP3A4 activity and thus accelerate the CYP3A4-mediated metabolism of ATZ (**Figures 2** and **5**).

Furthermore, ATZ and its metabolites, DEA and DIA, all underwent glutathione (GSH) conjugation in human liver microsomes and human liver S9 fractions (66). Studies showed that curcumin helped maintain and replenish the GSH pool as well as stimulated glutathione-*S*-transferase (GST) activity (64, 67). Male Sprague-Dawley rats treated with either turmeric (curcumin being the principal active ingredient) at doses of 100, 200, or 300 mg/kg body weight, or curcumin at 200 mg/kg, maintained baseline GSH levels despite receiving cytotoxic doses of CCl_4_, which is a hepatic toxicant that causes oxidative stress in the liver (64). Curcumin concentrations of 5-100 µM increased GST activity in LNCaP prostate cancer cells (67). In a preclinical study to investigate ATZ’s cardiotoxicity, rats orally exposed to ATZ dissolved in corn oil (to mimic human exposure) at 400 mg/kg per day for three weeks exhibited symptoms of oxidative stress in the heart and decreased GSH levels (70). Rats receiving the same ATZ exposure plus curcumin (400 mg/kg per day orally administered) had higher GSH levels, experienced less oxidative stress symptoms, and had overall improved condition of the heart (70). Based on the above findings in the heart, we propose that by ensuring GSH availability and stimulating GST, curcumin can promote ATZ metabolism and elimination and quench oxidative stress, not only in the heart, but also throughout the body, including the breast tissue, thereby mitigating ATZ’s toxicity (**Figure 5**). In summary, by reducing ATZ’s bioavailability, curcumin is capable of dampening ATZ’s overall toxicity in the body, including its promoting effect on breast cancer.

## Summary and Outlook

In the present review, by extracting existing qualitative data from the literature, we formulated a hypothesis aiming to explain the apparent connection between chronic exposure to ATZ and breast cancer development. In addition, we proposed possible mechanisms that may be responsible for the benefit of employing curcumin to counteract the effects of ATZ. Due to the nature of the review, we agree that studies using appropriate models are needed to test the hypotheses and mechanisms proposed. Further experimentation is needed to directly validate the benefits of curcumin counteracting the toxic effects of ATZ, specifically in the setting of breast cancer. Also, much of the evidence regarding ATZ and curcumin is from short-term studies (26, 70). There is a lack of long-term studies regarding these compounds, which limits our knowledge and application. We have proposed some long-term studies below, which will hopefully guide future research on ATZ and curcumin to produce needed data. It was reported that curcumin mitigated oxidative stress and cardiotoxicity caused by ATZ in rats (70). We suggest conducting a similar *in vivo* experiment in female rats with a focus on breast cancer outcomes. The dosing regimen of ATZ should be a wide range encompassing environmentally relevant exposure and also achieving toxic threshold, i.e., 0.4 mg/kg, 4 mg/kg, 40 mg/kg, and 400 mg/kg. The dosing regimen for curcumin will include (1) curcumin dissolved in corn oil and (2) curcumin formulated with piperine to enhance its bioavailability (19) (see discussion on increasing curcumin's bioavailability later in this section). In addition, instead of dosing for three weeks (70), the exposure duration would be six months or even longer, in accordance with the standard for chronic exposure studies in rodents (71), to mimic chronic exposure in humans. Our hypothesis is that the rats exposed to ATZ would develop more mammary tumors and/or at a faster rate than control rats (without ATZ exposure) after controlling for spontaneous mammary tumor rates. The rats exposed to ATZ and curcumin would develop fewer tumors and/or at a slower rate than those exposed to ATZ alone. Rats exposed to ATZ alone would also have higher estrogen levels and stronger estrogen signaling than the control rats and rats treated with ATZ and curcumin together. Measurable endpoints include the appearance of mammary tumors and estrogen levels in blood samples and in tumor biopsy tissue. We acknowledge that the above proposed study and some of the cited *in vivo* studies (26, 46, 64, 70) utilized high-dose exposure. It should be noted that this does not necessarily translate to chronic low-level human exposure as typically seen in the general population. Despite the marked differences between the doses used in the cited studies and realistic human exposure levels, high-dose toxicology studies have significant human relevance and have informed risk assessment (such as identifying potential hazards; determining the maximum tolerated dose, MTD; and establishing points of departure, PoD) (72, 73). Dose-response relationships established from such toxicology studies also bring tremendous value to our understanding of the mechanism of action toward the chemical of interest.

An *in vitro* study examining breast cancer cell proliferation under co-treatment with ATZ and curcumin will be worthwhile. To our knowledge, no studies have examined the effects of ATZ and curcumin administered together on human breast cancer cells. We propose an *in vitro* study to measure cell proliferation as well as EGF signaling activity in the presence of ATZ alone, EGFR antagonist alone, curcumin alone, ATZ and EGFR antagonist co-treatment, and ATZ and curcumin co-treatment. We predict that breast cancer cells treated with ATZ alone should have a greater proliferation rate associated with stronger EGF signaling activity, while the co-treatment of ATZ+antagonist and ATZ+Curcumin should produce limited proliferation and diminished EGF signaling activity. We also propose to measure CYP19A1 activity in the above treatment groups. We hypothesize that ATZ-alone treatment would increase CYP19A1 activity and estrogen levels (41, 43, 74). However, the increase in estrogen levels should not be significant in the co-treatment of ATZ+curcumin due to the increased degradation of estrogen caused by curcumin’s activation of CYP3A4, partial suppression of CYP19A1 by curcumin via miR-125a’s translational suppression of ERRα, and enhanced metabolism of ATZ by curcumin. Therefore, relevant endpoints to be measured include activities of CYP19A1 and CYP3A4 and levels of miR-125a and estrogen. Should the actual experimental results match our speculation, they would provide evidence supporting the benefits of curcumin for breast cancer, at least with human breast cancer cell lines. With the currently available techniques, the employment of patient-derived breast cancer organoid models would be able to provide even more direct evidence to further support curcumin’s use as a treatment for breast cancer.

A determination of curcumin’s effect on miR-125a would shed light on a point of contention. As previously mentioned, curcumin activates miR-125a in human osteosarcoma cell lines (57), but another study showed that curcumin inhibited miR-125a expression in human nasopharyngeal cancer cell line HONE1 (75). To our knowledge, no study has tested curcumin’s effect on miR-125a in breast tissue. A study examining miR-125a found that its activation inhibited ER-positive breast cancer *in vitro* and *in vivo*, although this study was unrelated to curcumin (76). An *in vivo* study employing rodent models of breast cancer would provide some definite answers to whether curcumin has anti-tumor effects on breast cancer and whether miR-125a and/or ERRα play an important role in this process. Such information is valuable to public health as it would provide mechanistic evidence on curcumin as an adjuvant therapy for breast cancer.

While this review focuses on curcumin in relation to combatting the toxicities of ATZ, curcumin alone also influences estrogenic pathways (77). For example, in a preclinical *in vitro* study examining curcumin’s effects on endometriosis, curcumin was found to decrease levels of estradiol in human endometrial cells (78). Another *in vitro* study, using human hepatocellular carcinoma Hepa 1–6 cells, found that curcumin significantly increased estrogen receptor alpha (ERα) gene expression (79). In the same study, curcumin also produced anticancer effects including induction of apoptosis and inhibition of cell growth (79). One *in vivo* study reported anti-metastasis effects and anti-proliferative effects and of curcumin in mice injected with MDA-MB-231 human breast cancer cells (80). In summary, curcumin alone seems to have modulatory effects on estrogen pathways in specific settings. Furthermore, curcumin also possesses the ability to halt cancer progression via inducing apoptosis, inhibiting proliferation, and hindering metastasis. These findings are in line with the theme of this review that curcumin is a promising potential antidote for ATZ due to its ability to modulate estrogenic effects and anticancer properties.

Curcumin’s low bioavailability may hinder its utility. Studies have suggested methods to enhance curcumin’s bioavailability in the human body (19, 20). One technique is co-administration with piperine, another natural substance (19). In humans, co-administration of 2 g curcumin and 20 mg piperine increased curcumin’s bioavailability by 2000% (19). Other methods to increase curcumin’s bioavailability include using a curcumin phospholipid complex and using curcumin nanoparticles (20). Continued pharmaceutical research to improve curcumin’s bioavailability will broaden its therapeutic utility, including its ability to counteract ATZ’s toxic effects. It has been reported that, upon oral administration to rats, a significant increase in serum tetrahydrocurcumin levels was detected, indicating a rather efficient metabolism converting the parent compound to this major metabolite (81). It is not clear whether this is true in humans and how much tetrahydrocurcumin is contributing to the observed effect ascribed to the parent compound.

An updated epidemiological study exploring the correlation between ATZ exposure and breast cancer is also desirable. The limited available studies on this topic from the PubMed database include a 2011 review paper that relied on older datasets (25) and a 2017 longitudinal study utilizing samples from 1991-1992 (32). This highlights the need for more recent and comprehensive investigations. An especially valuable approach could be to compare populations (matched in age, ethnic background, education, socioeconomic status, with confounders including BMI, comorbidity, and family history of breast cancer accounted for) in Europe and the United States over the past twenty years. ATZ was banned in the European Union in 2003 (8), so if there is a discrepancy in breast cancer rates among women between the United States and Europe, ATZ exposure could be a significant contributing factor. Also, in a long-term study following up on the effect of *in-utero* ATZ exposure, instead of looking at the correlation between *in-utero* ATZ exposure and the age of menarche (**Figure 1D**) (32), the correlation between *in-utero* ATZ exposure and breast cancer should be examined.

**Figure 6:**
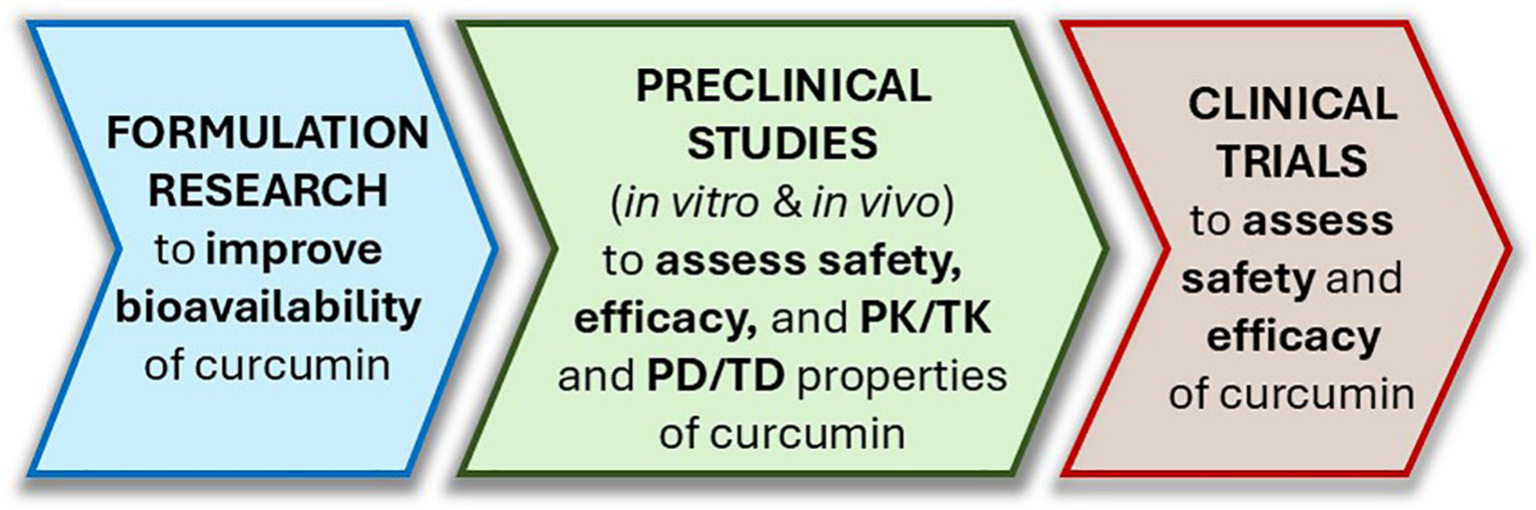
A roadmap outlining the steps leading curcumin from bench to bedside as an ATZ antidote. PK, pharmacokinetics; TK, toxicokinetics; PD, pharmacodynamics; TD, toxicodynamics.

In addition, studies aiming at establishing dose-response relationships of (1) ATZ on CYP19A1 induction/activity, (2) curcumin on ATZ detoxification with ATZ plasma level as the outcome measure, and (3) curcumin on counteracting ATZ’s promoting effect on breast cancer development with breast tissue proliferation index as the endpoint would be extremely valuable in supporting curcumin as a mechanism-based antidote for ATZ exposure. We have proposed a roadmap for curcumin outlining the key steps leading from its laboratory discoveries to potential clinical application (**Figure 6**).

The experiments suggested above should produce results with potentially significant public health implications. The findings could illuminate curcumin as a useful modality for breast cancer treatment and/or prevention, as curcumin is already available as a dietary supplement and abundantly present in the condiment turmeric. We would also learn more about the risk of ATZ exposure and its effects on breast cancer. This could facilitate risk assessment of ATZ and help make an informed decision on whether ATZ should be banned in the United States due to its potential to contribute to breast cancer risk.

## References

[1] World Cancer Research Fund. Breast cancer statistics. London: World Cancer Res. Fund (2025). Available online at: https://www.wcrf.org/preventing-cancer/cancer-statistics/breast-cancer-statistics/.

[2] Giaquinto AN Sung H Miller KD Kramer JL Newman LA Minihan A. Breast cancer statistics, 2022. CA Cancer J Clin. (2022) 72:524–41. doi: 10.3322/caac.21754, PMID: 36190501

[3] Caswell-Jin JL Sun LP Munoz D Lu Y Li Y Huang H. Analysis of breast cancer mortality in the US—1975 to 2019. JAMA. (2024) 331:233–41. doi: 10.1001/jama.2023.25881, PMID: 38227031 PMC10792466

[4] Calaf GM Ponce−Cusi R Aguayo F Muñoz JP Bleak TC. Endocrine disruptors from the environment affecting breast cancer (Review). Oncol Lett. (2020) 20:19–32. doi: 10.3892/ol.2020.11566, PMID: 32565930 PMC7286136

[5] Wan MLY Co VA El-Nezami H. Endocrine disrupting chemicals and breast cancer: a systematic review of epidemiological studies. Crit Rev Food Sci Nutr. (2022) 62:6549–76. doi: 10.1080/10408398.2021.1903382, PMID: 33819127

[6] Atwood D Paisley-Jones C. Pesticides Industry Sales and Usage: 2008–2012 Market Estimates. Washington, DC: US Environ. Prot. Agency (2017). Available online at: https://www.epa.gov/sites/default/files/2017-01/documents/pesticides-industry-sales-usage-2016_0.pdf.

[7] Ribaudo MO Bouzaher A. Atrazine: environmental characteristics and economics of management. (1994). doi: 10.22004/ag.econ.34011

[8] Rohr J. The atrazine saga and its importance to the future of toxicology, science, and environmental and human health. Environ Toxicol Chem. (2021) 40:1544–58. doi: 10.1002/etc.5037, PMID: 33999476

[9] US EPA. Atrazine (2025). Available online at: https://www.epa.gov/ingredients-used-pesticide-products/atrazine (Accessed May 27, 2025).

[10] Dai X-Y Lin J Zhu S-Y Guo J-Y Cui J-G Li J-L. Atrazine-induced oxidative damage via modulating xenobiotic-sensing nuclear receptors and cytochrome P450 systems in cerebrum and antagonism of lycopene. Food Chem Toxicol. (2022) 170:113462. doi: 10.1016/j.fct.2022.113462, PMID: 36216167

[11] McConnell LL Harman-Fetcho JA Iii JDH. Measured concentrations of herbicides and model predictions of atrazine fate in the patuxent river estuary. J Env QUAL. (2004) 33:594–604. doi: 10.2134/jeq2004.5940, PMID: 15074811

[12] The American Cancer Society medical and editorial content team. What Is Breast Cancer (2021). Available online at: https://www.cancer.org/cancer/types/breast-cancer/about/what-is-breast-cancer.html (Accessed October 11, 2024).

[13] The American Cancer Society medical and editorial content team. Types of Breast Cancer | About Breast Cancer (2021). Available online at: https://www.cancer.org/cancer/types/breast-cancer/about/types-of-breast-cancer.html (Accessed October 14, 2024).

[14] Hilton HN Clarke CL Graham JD. Estrogen and progesterone signalling in the normal breast and its implications for cancer development. Mol Cell Endocrinol. (2018) 466:2–14. doi: 10.1016/j.mce.2017.08.011, PMID: 28851667

[15] The American Cancer Society medical and editorial content team. Breast Cancer Hormone Receptor Status | Estrogen Receptor (2021). Available online at: https://www.cancer.org/cancer/types/breast-cancer/understanding-a-breast-cancer-diagnosis/breast-cancer-hormone-receptor-status.html (Accessed November 11, 2024).

[16] Gupta SC Patchva S Aggarwal BB. Discovery of curcumin, a component of golden spice, and its miraculous biological activities. Clin Exp Pharmacol Physiol. (2012) 39:283–99. doi: 10.1111/j.1440-1681.2011.05648.x, PMID: 22118895 PMC3288651

[17] Kocaadam B Şanlier N. Curcumin, an active component of turmeric ( *Curcuma longa* ), and its effects on health. Crit Rev Food Sci Nutr. (2017) 57:2889–95. doi: 10.1080/10408398.2015.1077195, PMID: 26528921

[18] Hewlings SJ Kalman DS. Curcumin: A review of its effects on human health. Foods Basel Switz. (2017) 6:92. doi: 10.3390/foods6100092, PMID: 29065496 PMC5664031

[19] Shoba G Joy D Joseph T Majeed M Rajendran R Srinivas PSSR. Influence of piperine on the pharmacokinetics of curcumin in animals and human volunteers. Planta Med. (2007) 64:353–6. doi: 10.1055/s-2006-957450, PMID: 9619120

[20] Anand P Kunnumakkara AB Newman RA Aggarwal BB. Bioavailability of curcumin: problems and promises. Mol Pharm. (2007) 4:807–18. doi: 10.1021/mp700113r, PMID: 17999464

[21] Human Metabolome Database: Showing metabocard for Atrazine (HMDB0041830) (2022). Available online at: https://hmdb.ca/metabolites/HMDB0041830 (Accessed October 14, 2024).

[22] McBirney M King SE Pappalardo M Houser E Unkefer M Nilsson E. Atrazine induced epigenetic transgenerational inheritance of disease, lean phenotype and sperm epimutation pathology biomarkers. PloS One. (2017) 12:e0184306. doi: 10.1371/journal.pone.0184306, PMID: 28931070 PMC5606923

[23] Abarikwu SO Costa GMJ de Lima e Martins Lara N Lacerda SMSN de França LR. Atrazine impairs testicular function in BalB/c mice by affecting Leydig cells. Toxicology. (2021) 455:152761. doi: 10.1016/j.tox.2021.152761, PMID: 33766575

[24] Cooksey C. Turmeric: old spice, new spice: Biotechnic & Histochemistry: Vol 92, No 5. Biotech Histochem. (2017) 92:309–14. doi: 10.1080/10520295.2017.1310924, PMID: 28506084

[25] Simpkins JW Swenberg JA Weiss N Brusick D Eldridge JC Stevens JT. Atrazine and breast cancer: A framework assessment of the toxicological and epidemiological evidence. Toxicol Sci. (2011) 123:441–59. doi: 10.1093/toxsci/kfr176, PMID: 21768606 PMC3179673

[26] Wang M Chen J Zhao S Zheng J He K Liu W. Atrazine promotes breast cancer development by suppressing immune function and upregulating MMP expression. Ecotoxicol Environ Saf. (2023) 253:114691. doi: 10.1016/j.ecoenv.2023.114691, PMID: 36868036

[27] Stevens JT Breckenridge CB Wetzel LT Gillis JH Luempert LG III Eldridge JC. Hypothesis for mammary tumorigenesis in Sprague-Dawley rats exposed to certain triazine herbicides. J Toxicol Environ Health. (1994) 43:139–53. doi: 10.1080/15287399409531911, PMID: 7932845

[28] Lasserre J-P Fack F Revets D Planchon S Renaut J Hoffmann L. Effects of the endocrine disruptors atrazine and PCB 153 on the protein expression of MCF-7 human cells. J Proteome Res. (2009) 8:5485–96. doi: 10.1021/pr900480f, PMID: 19778091

[29] Egiebor E. Cell proliferative and cell cycle effects of atrazine using human breast cell lines. Am J Biomed Sci Res. (2019) 4:421–6. doi: 10.34297/AJBSR.2019.04.000846

[30] Huang P Yang J Ning J Wang M Song Q. Atrazine triggers DNA damage response and induces DNA double-strand breaks in MCF-10A cells. Int J Mol Sci. (2015) 16:14353–68. doi: 10.3390/ijms160714353, PMID: 26114388 PMC4519846

[31] Stueve TR. Conserved Molecular and Epigenetic Determinants of Aromatase Gene Induction by the Herbicide Atrazine in Human and Rat Cellular Models Relevant to Breast Cancer Risk. Berkeley: University of California (2011). Available online at: https://escholarship.org/uc/item/2ft8q7t5 (Accessed March 19, 2025).

[32] Namulanda G Taylor E Maisonet M Barr DB Flanders WD Olson D. *In utero* exposure to atrazine analytes and early menarche in the Avon Longitudinal Study of Parents and Children Cohort. Environ Res. (2017) 156:420–5. doi: 10.1016/j.envres.2017.04.004, PMID: 28410519 PMC5679269

[33] Ritte R Tikk K Lukanova A Tjønneland A Olsen A Overvad K. Reproductive factors and risk of hormone receptor positive and negative breast cancer: a cohort study. BMC Cancer. (2013) 13:584. doi: 10.1186/1471-2407-13-584, PMID: 24321460 PMC3866571

[34] Li K Anderson G Viallon V Arveux P Kvaskoff M Fournier A. Risk prediction for estrogen receptor-specific breast cancers in two large prospective cohorts. Breast Cancer Res. (2018) 20:147. doi: 10.1186/s13058-018-1073-0, PMID: 30509329 PMC6276150

[35] E.H. and B.C.C. Group. Circulating sex hormones and breast cancer risk factors in postmenopausal women: reanalysis of 13 studies. Br J Cancer. (2011) 105:709. doi: 10.1038/bjc.2011.254, PMID: 21772329 PMC3188939

[36] Blanck HM Marcus M Tolbert PE Rubin C Henderson AK Hertzberg VS. Age at menarche and tanner stage in girls exposed *in utero* and postnatally to polybrominated biphenyl. Epidemiology. (2000) 11:641. doi: 10.1097/00001648-200011000-00005, PMID: 11055623

[37] Vasiliu O Muttineni J Karmaus W. *In utero* exposure to organochlorines and age at menarche. Hum Reprod. (2004) 19:1506–12. doi: 10.1093/humrep/deh292, PMID: 15131079

[38] Simpson ER Clyne C Rubin G Boon WC Robertson K Britt K. Aromatase—A brief overview. Annu Rev Physiol. (2002) 64:93–127. doi: 10.1146/annurev.physiol.64.081601.142703, PMID: 11826265

[39] Czajka-Oraniec I Simpson ER. Aromatase research and its clinical significance. Endokrynol Pol. (2010) 61:126–34., PMID: 20205115

[40] Fan W Yanase T Morinaga H Gondo S Okabe T Nomura M. Atrazine-induced aromatase expression is SF-1 dependent: implications for endocrine disruption in wildlife and reproductive cancers in humans. Environ Health Perspect. (2007) 115:720–7. doi: 10.1289/ehp.9758, PMID: 17520059 PMC1867956

[41] Fan W Yanase T Morinaga H Gondo S Okabe T Nomura M. Herbicide atrazine activates SF-1 by direct affinity and concomitant co-activators recruitments to induce aromatase expression via promoter II. Biochem Biophys Res Commun. (2007) 355:1012–8. doi: 10.1016/j.bbrc.2007.02.062, PMID: 17331471

[42] Kucka M Pogrmic-Majkic K Fa S Stojilkovic SS Kovacevic R. Atrazine acts as an endocrine disrupter by inhibiting cAMP-specific phosphodiesterase-4. Toxicol Appl Pharmacol. (2012) 265:19–26. doi: 10.1016/j.taap.2012.09.019, PMID: 23022511 PMC4181665

[43] Roberge M Hakk H Larsen G. Atrazine is a competitive inhibitor of phosphodiesterase but does not affect the estrogen receptor. Toxicol Lett. (2004) 154:61–8. doi: 10.1016/j.toxlet.2004.07.005, PMID: 15475179

[44] Stocco C. Aromatase expression in the ovary: hormonal and molecular regulation. Steroids. (2008) 73:473. doi: 10.1016/j.steroids.2008.01.017, PMID: 18321551 PMC2365984

[45] Liu D Yang M Zhu H Zheng Y Zhu X. Human pregnane X receptor-mediated transcriptional regulation of cytochrome P450 3A4 by some phytochemicals. Zhejiang Xue Xue Bao Yi Xue Ban J Zhejiang Univ Med Sci. (2006) 35:8–13. doi: 10.3785/j.issn.1008-9292.2006.01.002, PMID: 16470913

[46] Hsieh Y-W Huang C-Y Yang S-Y Peng Y-H Yu C-P Chao P-DL. Oral intake of curcumin markedly activated CYP 3A4: *in vivo* and ex-vivo studies. Sci Rep. (2014) 4:6587. doi: 10.1038/srep06587, PMID: 25300360 PMC5377466

[47] Bansal SS Kausar H Vadhanam MV Ravoori S Gupta RC. Controlled systemic delivery by polymeric implants enhances tissue and plasma curcumin levels compared with oral administration. Eur J Pharm Biopharm. (2012) 80:571–7. doi: 10.1016/j.ejpb.2011.12.009, PMID: 22227368 PMC3345811

[48] Li H Fu Y Gong W Wang G Li Z Tian J. Remission of copper-induced liver injury through the PXR/NF-kB signaling pathway: The effects of dietary curcumin supplementation in largemouth bass (*Micropterus salmoides*). Ecotoxicol Environ Saf. (2024) 285:117070. doi: 10.1016/j.ecoenv.2024.117070, PMID: 39317076

[49] Kluth D Banning A Paur I Blomhoff R Brigelius-Flohé R. Modulation of pregnane X receptor-and electrophile responsive element-mediated gene expression by dietary polyphenolic compounds. Free Radic Biol Med. (2007) 42:315–25. doi: 10.1016/j.freeradbiomed.2006.09.028, PMID: 17210444

[50] Nones K Dommels YEM Martell S Butts C McNabb WC Park ZA. The effects of dietary curcumin and rutin on colonic inflammation and gene expression in multidrug resistance gene-deficient ( *mdr1a* ^–/–^ ) mice, a model of inflammatory bowel diseases. Br J Nutr. (2008) 101:169–81. doi: 10.1017/S0007114508009847, PMID: 18761777

[51] Bartonkova I Dvorak Z. Essential oils of culinary herbs and spices activate PXR and induce CYP3A4 in human intestinal and hepatic *in vitro* models. Toxicol Lett. (2018) 296:1–9. doi: 10.1016/j.toxlet.2018.07.023, PMID: 30071242

[52] Klyushova LS Perepechaeva ML Grishanova AY. The role of CYP3A in health and disease. Biomedicines. (2022) 10:2686. doi: 10.3390/biomedicines10112686, PMID: 36359206 PMC9687714

[53] Yamazaki H Shaw PM Guengerich FP Shimada T. Roles of cytochromes P450 1A2 and 3A4 in the oxidation of estradiol and estrone in human liver microsomes. Chem Res Toxicol. (1998) 11:659–65. doi: 10.1021/tx970217f, PMID: 9625734

[54] Yaghjyan L Colditz GA. Estrogens in the breast tissue: a systematic review. Cancer Causes Control. (2011) 22:529–40. doi: 10.1007/s10552-011-9729-4, PMID: 21286801 PMC3652894

[55] Yang C Zhou D Chen S. Modulation of aromatase expression in the breast tissue by ERR1 orphan receptor. Cancer Res. (1999) 58:5695–700., PMID: 9865725

[56] Lu D Kiriyama Y Lee K Giguère V. Transcriptional regulation of the estrogen-inducible pS2 breast cancer marker gene by the ERR family of orphan nuclear receptors. Cancer Res. (2001) 61:6755–61., PMID: 11559547

[57] Chen P Wang H Yang F Chen H He W Wang J. Curcumin promotes osteosarcoma cell death by activating miR-125a/ERRα Signal pathway. J Cell Biochem. (2016) 118:74–81. doi: 10.1002/jcb.25612, PMID: 27231954

[58] Sigismund S Avanzato D Lanzetti L. Emerging functions of the EGFR in cancer. Mol Oncol. (2018) 12:3–20. doi: 10.1002/1878-0261.12155, PMID: 29124875 PMC5748484

[59] Oda K Matsuoka Y Funahashi A Kitano H. A comprehensive pathway map of epidermal growth factor receptor signaling. Mol Syst Biol. (2005) 1:2005. doi: 10.1038/msb4100014, PMID: 16729045 PMC1681468

[60] Levy DE Lee C. What does Stat3 do? J Clin Invest. (2002) 109:1143–8. doi: 10.1172/JCI15650, PMID: 11994402 PMC150972

[61] Albanito L Lappano R Madeo A Chimento A Prossnitz ER Cappello AR. Effects of atrazine on estrogen receptor α– and G protein–coupled receptor 30–mediated signaling and proliferation in cancer cells and cancer-associated fibroblasts. Environ Health Perspect. (2015) 123:493–9. doi: 10.1289/ehp.1408586, PMID: 25616260 PMC4421771

[62] Starok M Preira P Vayssade M Haupt K Salomé L Rossi C. EGFR inhibition by curcumin in cancer cells: A dual mode of action. Biomacromolecules. (2015) 16:1634–42. doi: 10.1021/acs.biomac.5b00229, PMID: 25893361

[63] Zhen L Fan D Yi X Cao X Chen D Wang L. Curcumin inhibits oral squamous cell carcinoma proliferation and invasion via EGFR signaling pathways. Int J Clin Exp Pathol. (2014) 7:6438., PMID: 25400722 PMC4230161

[64] Lee H-Y Kim S-W Lee G-H Choi M-K Jung H-W Kim Y-J. Turmeric extract and its active compound, curcumin, protect against chronic CCl4-induced liver damage by enhancing antioxidation, BMC Complement. Altern Med. (2016) 16:316. doi: 10.1186/s12906-016-1307-6, PMID: 27561811 PMC5000414

[65] Robin SKD Ansari M Uppugunduri CRS. Spectrophotometric screening for potential inhibitors of cytosolic glutathione S-transferases. J Vis Exp. (2020) 2020(164):1–18. doi: 10.3791/61347, PMID: 33104076

[66] Joo H Choi K Hodgson E. Human metabolism of atrazine. Pestic Biochem Physiol - PESTIC Biochem Physiol. (2010) 98:73–9. doi: 10.1016/j.pestbp.2010.05.002

[67] Dubey V Owusu-Apenten R. Curcumin restores glutathione-S-transferase activity for LNCaP prostate cancer cells. Pure Appl Chem Sci. (2014) 2:61–72. doi: 10.12988/pacs.2014.411

[68] Bohn T Cocco E Gourdol L Guignard C Hoffmann L. Determination of atrazine and degradation products in Luxembourgish drinking water: origin and fate of potential endocrine-disrupting pesticides. Food Addit Contam Part A. (2011) 28:1041–54. doi: 10.1080/19440049.2011.580012, PMID: 21707270

[69] Migeot V Albouy-Llaty M Carles C Limousi F Strezlec S Dupuis A. Drinking-water exposure to a mixture of nitrate and low-dose atrazine metabolites and small-for-gestational age (SGA) babies: A historic cohort study. Environ Res. (2013) 122:58–64. doi: 10.1016/j.envres.2012.12.007, PMID: 23340115

[70] Keshk W Soliman N Mohamed Abo El-Noor M Wahdan A Shareef M. Modulatory effects of curcumin on redox status, mitochondrial function, and caspace-3 expression during atrazin-induced toxicity. J Biochem Mol Toxicol. (2014) 28:378–85. doi: 10.1002/jbt.21574, PMID: 24863870

[71] Pawar B Gupta T Vasdev N Tekade M Arafat B Tekade RK. Chapter 1 - Understanding pharmacotoxicology. In: Tekade R, editor. Essent. Pharmatoxicology Drug Res. Saudi Arabia: Academic Press (2023). p. 1–28. doi: 10.1016/B978-0-443-15840-7.00025-7

[72] Woutersen M Muller A Pronk MEJ Cnubben NHP Hakkert BC. Regulating human safety: How dose selection in toxicity studies impacts human health hazard assessment and subsequent risk management options. Regul Toxicol Pharmacol. (2020) 114:104660. doi: 10.1016/j.yrtph.2020.104660, PMID: 32334039

[73] Borgert CJ Fuentes C Burgoon LD. Principles of dose-setting in toxicology studies: the importance of kinetics for ensuring human safety. Arch Toxicol. (2021) 95:3651–64. doi: 10.1007/s00204-021-03155-4, PMID: 34623454 PMC8536606

[74] Holloway AC Anger DA Crankshaw DJ Wu M Foster WG. Atrazine-induced changes in aromatase activity in estrogen sensitive target tissues. J Appl Toxicol. (2008) 28:260–70. doi: 10.1002/jat.1275, PMID: 17685393

[75] Gao W Chan JY-W Wong T-S. Curcumin exerts inhibitory effects on undifferentiated nasopharyngeal carcinoma by inhibiting the expression of miR-125a-5p. Clin Sci. (2014) 127:571–9. doi: 10.1042/CS20140010, PMID: 24896104

[76] Zheng L Meng X Li X Zhang Y Li C Xiang C. miR-125a-3p inhibits ERα transactivation and overrides tamoxifen resistance by targeting CDK3 in estrogen receptor-positive breast cancer. FASEB J Off Publ Fed Am Soc Exp Biol. (2018) 32:588–600. doi: 10.1096/fj.201700461RR, PMID: 28939591

[77] Bachmeier BE Mirisola V Romeo F Generoso L Esposito A Dell’Eva R. Reference profile correlation reveals estrogen-like trancriptional activity of curcumin. Cell Physiol Biochem. (2010) 26:471–82. doi: 10.1159/000320570, PMID: 20798532

[78] Zhang Y Cao H Yu Z Peng H-Y Zhang C. Curcumin inhibits endometriosis endometrial cells by reducing estradiol production. Iran J Reprod Med. (2013) 11:415–22., PMID: 24639774 PMC3941414

[79] Sanaei M Kavoosi F Arabloo M. Effect of Curcumin in Comparison with Trichostatin A on the Reactivation of Estrogen Receptor Alpha gene Expression, Cell Growth Inhibition and Apoptosis Induction in Hepatocellular Carcinoma Hepa 1–6 Cell lLine. Asian Pac J Cancer Prev APJCP. (2020) 21:1045–50. doi: 10.31557/APJCP.2020.21.4.1045, PMID: 32334468 PMC7445996

[80] Bachmeier B Nerlich A Iancu C Cilli M Schleicher E Vené R. The chemopreventive polyphenol curcumin prevents hematogenous breast cancer metastases in immunodeficient mice. Cell Physiol Biochem. (2007) 19:137–52. doi: 10.1159/000099202, PMID: 17310108

[81] Inano H Onoda M Inafuku N Kubota M Kamada Y Osawa T. Chemoprevention by curcumin during the promotion stage of tumorigenesis of mammary gland in rats irradiated with γ-rays. Carcinogenesis. (1999) 20:1011–8. doi: 10.1093/carcin/20.6.1011, PMID: 10357781

